# Time course of altered DNA methylation evoked by critical illness and by early administration of parenteral nutrition in the paediatric ICU

**DOI:** 10.1186/s13148-020-00947-w

**Published:** 2020-10-20

**Authors:** Ines Verlinden, Fabian Güiza, Inge Derese, Pieter J. Wouters, Koen Joosten, Sascha C. Verbruggen, Greet Van den Berghe, Ilse Vanhorebeek

**Affiliations:** 1 Clinical Division and Laboratory of Intensive Care Medicine, Department of Cellular and Molecular Medicine, KU Leuven, Herestraat 49, 3000 Leuven, Belgium; 2 Intensive Care Unit, Department of Paediatrics and Paediatric Surgery, Erasmus Medical Centre, Sophia Children’s Hospital, Rotterdam, The Netherlands

**Keywords:** Paediatric intensive care unit, PICU, Critical illness, Nutrition, Parenteral nutrition, DNA methylation, Epigenetics, Time, Time course

## Abstract

**Background:**

A genome-wide study identified *de novo* DNA methylation alterations in leukocytes of children at paediatric intensive care unit (PICU) discharge, offering a biological basis for their impaired long-term development. Early parenteral nutrition (early-PN) in PICU, compared with omitting PN in the first week (late-PN), explained differential methylation of 23% of the affected CpG-sites. We documented the time course of altered DNA methylation in PICU and the impact hereon of early nutritional management.

**Results:**

We selected 36 early-PN and 36 late-PN matched patients, and 42 matched healthy children. We quantified DNA methylation on days 3, 5 and 7 for the 147 CpG-sites of which methylation was normal upon PICU admission in this subset and altered by critical illness at PICU discharge. Methylation in patients differed from healthy children for 64.6% of the 147 CpG-sites on day 3, for 72.8% on day 5 and for 90.5% on day 7 as revealed by ANOVA at each time point. Within-patients methylation time course analyses for each CpG-site identified different patterns based on paired *t* test *p* value and direction of change. Rapid demethylation from admission to day 3 occurred for 76.2% of the CpG-sites, of which 67.9% remained equally demethylated or partially remethylated and 32.1% further demethylated beyond day 3. From admission to day 3, 19.7% of the CpG-sites became hypermethylated, of which, beyond day 3, 34.5% remained equally hypermethylated or partially demethylated again and 65.5% further hypermethylated. For 4.1% of the CpG-sites, changes only appeared beyond day 3. Finally, for the CpG-sites affected by early-PN on the last PICU day, earlier changes in DNA methylation were compared for early-PN and late-PN patients, revealing that 38.9% were already differentially methylated by day 3, another 25.0% by day 5 and another 13.9% by day 7.

**Conclusions:**

Critical illness- and early-PN-induced changes in DNA methylation occurred mainly within 3 days. Most abnormalities were at least partially maintained or got worse with longer time in PICU. Interventions targeting aberrant DNA methylation changes should be initiated early.

## Background

Children who have been critically ill and required treatment in the paediatric intensive care unit (PICU) often suffer from adverse long-term health and developmental sequelae that are present years after hospital discharge [1]. Impaired neurocognitive development has been most thoroughly documented, but also growth retardation, poor physical functioning and long-term health risks have been reported [1–4]. Part of this long-term legacy appears preventable through altering aspects of the intensive care management. For example, nutritional management during critical illness was found to modify not only short-term outcome of critically ill children, but also the long-term legacy [5–9]. Indeed, providing full nutritional intake early with the use of parenteral nutrition (PN) to supplement insufficient enteral nutrition (early-PN) has shown to be clinically inferior than accepting the early macronutrient deficit by postponing any PN to beyond the first week in PICU (late-PN). Early-PN not only caused more infections and delayed recovery from the illness, it also prevented normal development of executive functions and/or caused behavioural problems, as assessed 2 and 4 years later [5, 8, 9].

We have recently provided evidence for a molecular basis of the long-term developmental legacy of critical illness and of the role of early-PN herein. In a large genome-wide study, we have shown that critical illness and the administration of early-PN during critical illness altered methylation of DNA extracted from peripheral blood of these young patients [10]. More specifically, at the day of PICU discharge, we documented alterations in methylation of several CpG-sites, changes that were not yet present upon PICU admission. Several of the affected CpG-sites were located in genes involved in a variety of relevant molecular functions, physical and neurocognitive developmental processes and in neurodegenerative and psychiatric diseases. The use of early-PN explained 23% of these *de novo* changes in DNA methylation. Importantly, these changes in DNA methylation statistically explained an important part of the negative impact of critical illness and of early-PN on neurocognitive development 2 years later.

The previous work thus revealed that DNA methylation is altered by critical illness and/or its intensive care management. However, it remained unclear how fast these changes in DNA methylation occur and how they evolve over time in the PICU. In a subset of matched patients and controls, we therefore documented the time course in PICU of the DNA methylation status of the CpG-sites of which methylation status was normal upon PICU admission, but previously shown to become aberrant at the day of PICU discharge [10], and studied the impact hereon of the use of early-PN versus late-PN.

## Results

### Participant characteristics

Demographics of the 42 healthy children and demographics and medical characteristics of the 72 patients upon PICU admission selected for this study are shown in Table 1. Total energy intake of patients was higher in the early-PN group than in the late-PN group on each of the first 7 days in PICU (Additional file 3).

**Table 1:** Demographics of participants and medical characteristics of patients upon PICU admission

	PICU patients	Healthy controls	*p* value	PICU patients Early-PN	PICU patients Late-PN	*p* value
	*n* = 42	*n* = 42		*n* = 36	*n* = 36	
*Demographics*						
Age (mean ± SEM)—years	3.9 ± 0.6	3.8 ± 0.5	0.93	2.6 ± 0.7	2.1 ± 0.5	0.57
Infant (age < 1 year)—no. (%)	14 (33)	10 (24)	0.33	22 (61)	22 (61)	1.00
Sex			0.82			0.81
Female—no. (%)	21 (50)	22 (48)		15 (42)	14 (39)	
Male—no. (%)	21 (50)	20 (52)		21 (58)	22 (61)	
Socio-economic status^a^						
Educational level of the parents			–			0.13
Parents educational level 1.5—no. (%)	3 (7)	NA		4 (11)	1 (3)	
Parents educational level 2—no. (%)	10 (24)	NA		11 (31)	9 (25)	
Parents educational level 2.5—no. (%)	6 (14)	NA		4 (11)	7 (19)	
Parents educational level 3—no. (%)	10 (24)	NA		6 (17)	13 (36)	
Parents educational level unknown—no. (%)	13 (31)	NA		11 (31)	6 (17)	
Occupational level of the parents			–			0.28
Parents occupational level 1.5—no. (%)	2 (5)	NA		2 (6)	2 (6)	
Parents occupational level 2—no. (%)	8 (19)	NA		8 (22)	8 (22)	
Parents occupational level 2.5—no. (%)	2 (5)	NA		2 (6)	3 (8)	
Parents occupational level 3—no. (%)	7 (17)	NA		5 (14)	9 25)	
Parents occupational level 3.5—no. (%)	1 (2)	NA		1 (3)	0 (0)	
Parents occupational level 4—no. (%)	5 (12)	NA		3 (8)	7 (19)	
Parents occupational level unknown—no. (%)	17 (40)	NA		15 (41)	7 (19)	
*Patient characteristics upon PICU admission*						
Weight z-score (mean ± SEM)	− 0.45 ± 0.23	NA	–	− 0.48 ± 0.21	− 0.41 ± 0.25	0.84
Height z-score (mean ± SEM)	− 0.63 ± 0.34	NA	–	− 0.61 ± 0.28	− 0.21 ± 0.36	0.39
STRONGkids risk level^b^			–			0.15
Medium—no. (%)	38 (90)	NA		35 (97)	32 (89)	
High—no. (%)	4 (10)	NA		1 (3)	4 (11)	
PeLOD score first 24 h in PICU (mean ± SEM)^c^	28.3 ± 1.4	NA	–	29.4 ± 1.5	30.0 ± 1.2	0.72
PIM3 score (mean ± SEM)^d^	− 3.06 ± 0.17	NA	–	− 2.87 ± 0.20	− 3.23 ± 0.12	0.13
Infection upon PICU admission	14 (33)	NA	–	9 (25)	9 (25)	1.00
Diagnostic category^e^		NA	–			0.23
Surgical-cardiac—no. (%)	23 (55)	NA		24 (67)	27 (75)	
Surgical-other—no. (%)	4 (10)	NA		3 (8)	2 (6)	
Neurosurgery/neurology—no. (%)	6 (14)	NA		4 (11)	2 (6)	
Trauma/burn—no. (%)	1 (2)	NA		0 (0)	1 (3)	
Transplantation/haematology/oncology—no. (%)	2 (5)	NA		1 (3)	1 (3)	
Medical-other—no. (%)	6 (14)	NA		4 (11)	3 (8)	
History of malignancy—no. (%)	5 (12)	NA	–	3 (8)	2 (6)	0.64
Diabetes—no. (%)	0 (0)	NA	–	0 (0)	0 (0)	–
Predefined syndrome—no. (%)^f^	8 (19)	NA	–	8 (22)	3 (8)	0.09
*Randomisation*						
Early-PN—no. (%)	19 (45)	NA	–	36 (100)	0 (0)	–
Late-PN—no. (%)	23 (55)	NA	–	0 (0)	36 (100)	–

*PN* parenteral nutrition, *SEM* standard error of the mean, *PICU* paediatric intensive care unit, *STRONGkids* screening tool for risk on nutritional status and growth, *PeLOD* paediatric logistic organ dysfunction, *PIM3* paediatric index of mortality 3

^a^The educational and occupational level is the mean of the paternal and maternal educational or occupational level (Additional file 1)

^b^STRONGkids scores range from 0 to 5, with a score of 0 indicating a low risk of malnutrition, a score of 1–3 indicating a medium risk, and a score of 4–5 indicating a high risk

^c^PeLOD scores range from 0 to 71, with higher scores indicating more severe illness

^d^Higher PIM3 scores indicate a higher risk of mortality

^e^‘Surgical-other’ includes abdominal, thoracic or, other surgery. ‘Medical-other’ includes cardiac, gastrointestinal or hepatic, renal, respiratory or, other medical problems

^f^A predefined syndrome is any pre-randomisation syndrome or illness a priori defined as affecting or possibly affecting neurocognitive development (Additional file 2)

### Studied CpG-sites

We performed this study on the 147 CpG-sites of which methylation status was previously shown to be altered by critical illness and/or its intensive care management [10] but normal upon PICU admission in the current participants’ subset. For 36 of these 147 CpG-sites, the use of early-PN versus late-PN contributed to the differential methylation [10].

The studied CpG-sites are clinically relevant, since DNA methylation alterations that occur in these CpG-sites during PICU stay were shown to play a mediating role in the negative impact of critical illness and, for 36 of them of early-PN versus late-PN during critical illness, on the long-term neurocognitive development of children [10]. Location of the 147 studied CpG-sites and gene-related protein functions are shown in Additional file 4.

### DNA methylation alterations over time in 147 CpG-sites in critically ill patients versus healthy children

At PICU day 3, 64.6% of the CpG-sites already showed a significant difference in methylation status in patients as compared with healthy children (Additional file 5). This fraction increased to 72.8% by day 5 and to 90.5% by day 7.

### Pattern analysis of DNA methylation alterations over time in 147 CpG-sites affected by critical illness

**Fig. 1:**
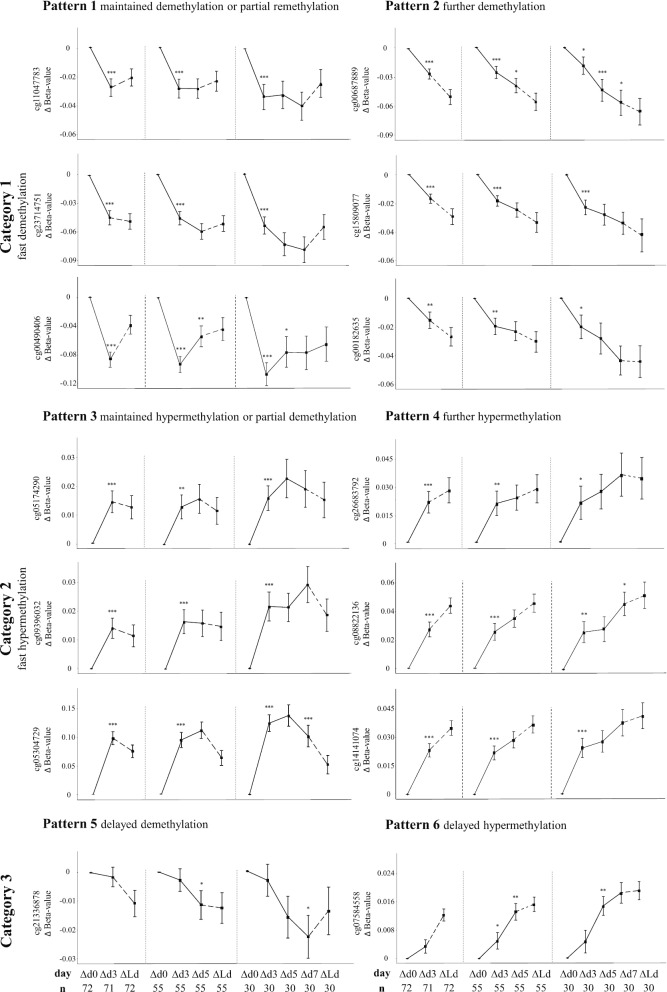
Evolution of DNA methylation over time in critically patients. Detailed time profiles are shown for 14 representative CpG-sites distributed over the identified patterns of changes in methylation status in PICU. Separate graphs are shown for patients in PICU for at least 3 days, at least 5 days, or at least 7 days. Data are presented as mean and standard error. *n* represents the number of patients with samples available at the respective time points. *p* values shown were obtained with paired *t* tests and represent the comparison with the previous time point. *p value ≤ 0.05, **p value ≤ 0.01, ***p value ≤ 0.001. Tests were not performed to compare the last PICU day with the previous time point, in view of the variability of the last day (which also was equal to day 3, day 5 or day 7 for some of the patients). Δd0: *β* value day 0 minus *β* value day 0, Δd3: *β* value day 3 minus *β* value day 0, Δd5: *β* value day 5 minus *β* value day 0, ∆d7: *β* value day 7 minus *β* value day 0, ΔLd: *β* value last PICU day minus *β* value day 0

The time series of the within-patient changes in DNA methylation revealed three major pattern categories between PICU admission and day 3, which could be further divided into six patterns based on the evolution beyond day 3. The detailed time courses of representative CpG-sites for each of these patterns are illustrated in Fig. 1 and Additional file 6. Additionally, heatmaps were constructed providing an overview of all CpG-sites of the four most abundant patterns (Additional file 7).

In the first pattern category, which covered the majority (76.2%) of the CpG-sites, rapid demethylation occurred between admission and day 3 (paired *t* test *p* < 0.05). Beyond day 3, 67.9% of these CpG-sites subsequently remained equally demethylated or only partially remethylated (pattern 1), whereas the other 32.1% further demethylated beyond day 3 (pattern 2), although mostly at a slower rate.

In the second pattern category, hypermethylation of 19.7% of the CpG-sites occurred between admission and day 3 (paired *t* test *p* < 0.05). Beyond day 3, 34.5% of these CpG-sites remained equally hypermethylated or only partially demethylated again (pattern 3), while the other 65.5% further hypermethylated beyond day 3 (pattern 4). The further hypermethylation mostly progressed at a slower rate than the initial rise towards day 3.

The third pattern category covered 4.1% of CpG-sites of which methylation did not change between admission and day 3 (paired *t* test *p* > 0.05) and for which clear changes became visible only beyond day 3. Of these CpG-sites, 50% showed a delayed demethylation (pattern 5) and 50% showed a delayed hypermethylation (pattern 6).

### Pattern analysis of DNA methylation alterations over time in 36 CpG-sites affected by early-PN versus late-PN during critical illness

Of the CpG-sites of which DNA methylation was previously shown to be affected by early-PN versus late-PN on the last PICU day [10], 33.3% were assigned to pattern 1 (rapid demethylation followed by equally maintained demethylation or only partial remethylation), 38.9% to pattern 2 (rapid demethylation followed by further demethylation), 5.6% to pattern 3 (rapid hypermethylation followed by equally maintained hypermethylation or only partial demethylation), and 22.2% to pattern 4 (rapid hypermethylation followed by even further hypermethylation).

**Fig. 2:**
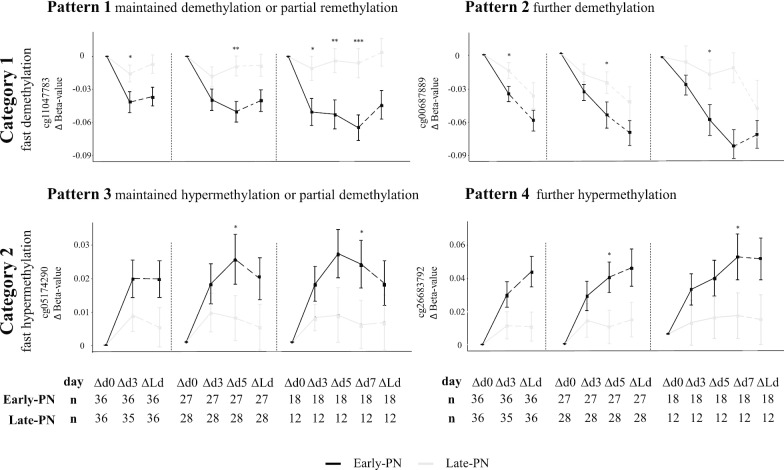
Evolution of DNA methylation over time in early-PN versus late-PN patients. Detailed time profiles are shown for four representative CpG-sites distributed over the four major identified patterns of changes in methylation status in PICU. Black lines represent the patients in the early-PN group, and grey lines represent patients in the late-PN group (pooled profiles for early-PN and late-PN patients are shown in Fig. 1). Separate graphs are shown for patients in PICU for at least 3 days, at least 5 days, or at least 7 days. Data are presented as mean and standard error. *n* represents the number of patients with samples available at the respective time points. *p* values shown were obtained with *t* tests and represent the comparison among patients in the early-PN and late-PN groups at day 3, day 5 or day 7. *p value ≤ 0.05, **p value ≤ 0.01, ***p value ≤ 0.001. Tests were not performed to compare early-PN and late-PN on the last PICU day, in view of the variability of the last day (which also was equal to day 3, day 5 or day 7 for some of the patients). Δd0: *β* value day 0 minus *β* value day 0, Δd3: *β* value day 3 minus *β* value day 0, Δd5: *β* value day 5 minus *β* value day 0, ∆d7: *β* value day 7 minus *β* value day 0, ΔLd: *β* value last PICU day minus *β* value day 0

The DNA methylation time course for representative CpG-sites of pattern 1 to 4 in early-PN versus late-PN patients is shown in Fig. 2. In late-PN patients, DNA methylation generally remained relatively stable with less obvious changes, whereas time-dependent patterns were observed in the early-PN patients. Of these CpG-sites affected by early-PN versus late-PN by PICU discharge, 38.9% became differentially methylated between early-PN and late-PN patients already on day 3, another 25.0% of these CpG-sites became differentially methylated by day 5 and another 13.9% by day 7**.**

## Discussion

This study aimed at visualising time courses and patterns of the previously identified abnormal DNA methylation brought about by critical illness, in part attributable to early use of PN. The study revealed that most abnormal DNA methylation occurred fast, most often within 3 days, and that most abnormalities were at least partially maintained or got worse over the first week in the PICU.

It is known that the epigenome has a great plasticity and undergoes physiological and disease-associated alterations in response to internal and external stimuli [11, 12]. Although studies on DNA methylation dynamics evaluating intraindividual longitudinal changes in DNA methylation are scarce, there is emerging evidence that an individual’s DNA methylation profile is subject to change on both a short- (hours to days) and long-term (months to years) timescale [13, 14]. For the majority of the CpG-sites investigated in the present study, the largest changes in DNA methylation status occurred within the first 3 days after PICU admission, whereas any further changes beyond day 3 progressed at a slower rate. Earlier research in other fields also documented short-term DNA methylation alterations in several CpG-sites, for instance within only hours after metformin administration [15], or within a few days after the start of a detoxification treatment in alcohol-dependent individuals [16]. Also, in physiologic conditions, DNA demethylation and methylation can occur rapidly, such as within a timeframe of several hours after fertilisation [17].

This study started from the previously identified *de novo* alterations in DNA methylation that arose after PICU admission and remained present until discharge from the PICU [10]. We distinguished different patterns among the time-dependent changes in methylation status of the studied CpG-sites during PICU stay. For several of the studied CpG-sites that underwent an initial rapid demethylation or hypermethylation, changes were at least partially maintained beyond day 3 in the PICU, whereas others continued along the line of further demethylation or hypermethylation. How long the identified DNA methylation alterations persist in children who have been critically ill remains to be investigated. Partial reversibility of DNA methylation would be consistent with previous findings on the evolution of tobacco smoking-related epigenetic changes after smoking cessation. Indeed, in former smokers, two classes of CpG-sites were described with, on the one hand, CpG-sites of which the methylation status reverted to levels of typical never smokers after smoking cessation and, on the other hand, CpG-sites that remained differentially methylated even up to more than 35 years after smoking cessation [18].

Multiple factors may contribute to the rapid changes in DNA methylation in critically ill children. Critical illness represents a severe physical stress, which rapidly activates a variety of neuronal, humoral and inflammatory pathways [19]. Activation of these pathways has been linked with several DNA methylation alterations and could thus play a role [20–22]. Also, nutrition can affect epigenetic mechanisms, directly via products of intermediary metabolism [23] or indirectly via the ability to activate several inflammatory pathways [24–26]. As described, early-PN as compared with late-PN independently contributed to the alterations in 23% of the CpG-sites that became differentially methylated by the last PICU day as compared with healthy children [10]. We have now shown that when comparing patients in the early-PN and late-PN group, the dynamic profiles of the changes in most of these CpG-sites already separated early within the first 3 to 5 days after PICU admission. DNA methylation in the late-PN patients in general remained relatively stable with less obvious changes, whereas the time-dependent patterns were mostly observed in the early-PN patients, suggesting that changes documented for the total group of critically ill children are mainly caused by the use of early-PN in these CpG-sites. The present data, showing that the aberrant DNA methylation changes that have previously been associated with impaired long-term developmental outcome of critically ill children [10] arise already very early after PICU admission, indicate that interventions aimed at the attenuation of such changes should be initiated from early onwards in the disease process. Withholding early-PN indeed partially prevented early aberrant changes in DNA methylation and partially protected against developmental impairments. This opens perspectives for other interventions that target the residual changes to further improve long-term developmental outcome of critically ill children.

Strengths of this study include the paired measurements per patient that allowed evaluation of within-patient evolution of DNA methylation status over time, the randomised controlled study design of the PEPaNIC trial, and the further building of this study on the previous rigorous, very stringent identification of CpG-sites affected by critical illness and early nutritional management in a large genome-wide DNA methylation study [10]. This study also has some weaknesses. First, the sample size of patients who were included in this time course study was substantially smaller as compared with the original study cohort, reducing statistical power. However, in this well-matched subset, within-patient changes could be analysed. Second, the classification of these within-patient DNA methylation time series into patterns was to a certain degree arbitrary. Third, except for the initial comparison of critically ill with healthy children, we did not correct for multiple testing. This was considered appropriate given that the aim of the second part of this study was to recognise patterns rather than absolute differences. Finally, we did not pursue mechanistic analyses beyond identifying the time course of DNA methylation changes.

## Conclusions

DNA methylation changes evoked by critical illness in children and by the administration of early-PN in PICU occurred rapidly, mostly within the first few days of PICU admission. With longer time spent in the PICU after these first days, most abnormalities in DNA methylation were at least partially maintained or got worse. Future interventions aimed at attenuating such aberrant changes in DNA methylation should be initiated early after PICU admission.

## Methods

### Study population and blood sampling

**Fig. 3:**
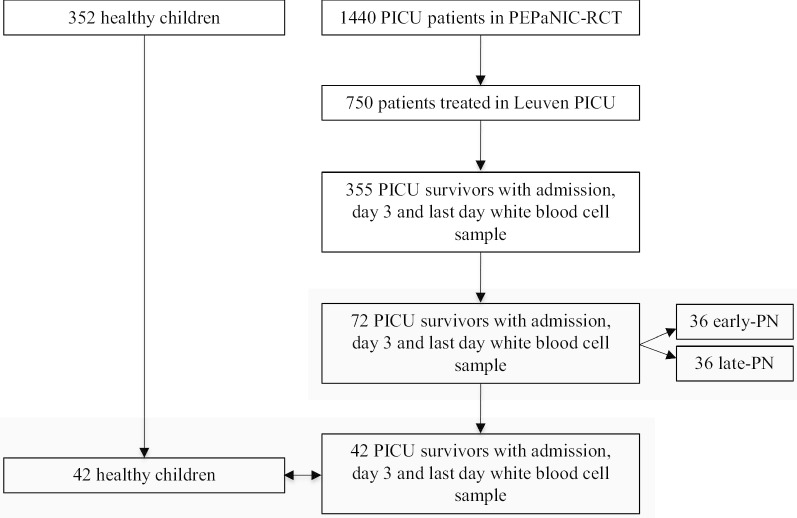
CONSORT diagram of study participants. PICU: paediatric intensive care unit, PEPaNIC: Paediatric Early versus Late Parenteral Nutrition in Intensive Care Unit, RCT: randomised controlled trial, PN: parenteral nutrition

This study is a preplanned secondary analysis of the multicentre PEPaNIC randomised controlled trial that included 1440 critically ill children aged 0–17 years admitted to the paediatric intensive care units of Leuven (Belgium), Rotterdam (The Netherlands) or Edmonton (Canada). Patients had been randomly assigned to early initiation of PN within 24 h when enteral nutrition was insufficient (early-PN), or to postponing any supplemental PN to beyond the first week in PICU (late-PN). After 1 week, for both groups equally, PN could be administered if necessary. The full study protocol and primary outcomes have been published [7]. Repeated blood cell samples, destined to the extraction of DNA, had been collected upon PICU admission, at day 3, day 5, and day 7, and at the last day in the PICU. For comparison with healthy control children, blood had been sampled from children who had never been admitted to a PICU (*n* = 352), immediately after placement of an intravenous catheter prior to minor elective surgery (Fig. 3) [10].

In order to document the time course of the *de novo* changes evoked by early-PN versus late-PN during PICU stay, in a financially feasible way and while avoiding confounders, a limited selection of well-matched patients was required (Table 1). To this end, we selected a subgroup of 36 late-PN and 36 early-PN patients matched for baseline risk factors from all Leuven patients who needed intensive care for more than 3 days, who were discharged alive from PICU and for whom DNA was available for the admission day, day 3 and last day in PICU (Fig. 3). This matching was performed with the use of propensity score matching [SPSS R-menu R3.1 (Foundation for Statistical Computing) in IBM SPSS Statistics 23.0.0.0 (SPSS, Chicago, IL)]. Logistic regression was used to estimate propensity scores with the baseline risk factors age, type (admission diagnosis) and severity of illness (PeLOD score), infection upon admission, gender, height, weight and educational level of the parents (Additional file 1) as covariates, and the caliper was set at 0.4. Of these 72 patients, 55 also had a day 5 sample (27 early-PN, 28 late-PN) and 30 had a day 7 sample (18 early-PN, 12 late-PN).

From these 72 patients, 42 (19 early-PN and 23 late-PN) could subsequently be propensity score-matched with 42 healthy control children with age and gender as covariates and caliper 0.2 (Fig. 3). Of these 42 patients, 34 had a day 5 sample (14 early-PN, 20 late-PN) and 18 had a day 7 sample (nine early-PN, nine late-PN).

### DNA methylation assessment

Genomic DNA was extracted from the blood cell samples and bisulphite-converted. DNA extractions were performed with the Maxwell®RSC instrument and the corresponding blood DNA purification kit (Promega Benelux b.v., Leiden, The Netherlands). All DNA concentrations were accurately quantified with the Qubit® 3.0 fluorometer (Thermo Fisher Scientific, Waltham, MA). Bisulphite conversion of the DNA (400 ng) was performed with the EZ DNA Methylation-Direct® Kit (Zymo Research, Irvine, CA). For one PICU-day 3 sample, DNA yield was insufficient for further analysis.

A genome-wide DNA methylation analysis of all samples was performed with the Infinium® HumanMethylation EPIC BeadChip (Illumina Inc., San Diego, CA), interrogating > 850.000 CpG-sites per sample and spanning > 99% of genes in the Reference Sequence (RefSeq) database [27].

Methylation data were preprocessed using Partek Genomics Suite® 7.0 (Partek, St. Louis, MO). Methylation *β* values ranging from 0 (no methylation) to 1 (full methylation) were obtained after background and functional normalisation of the raw intensities (i.e. an extension to quantile normalisation that removes unwanted technical variation using control probes). The data were subsequently subjected to several quality assessments at sample and probe level. First, sample histogram formation assessed the bipeak curve of the *β* value distribution among the samples in the low- and high-end range, and principal component analysis (PCA) assessed the variance in the dataset [10]. Next, of the 159 CpG-sites that we had previously identified in a genome-wide DNA methylation study as affected by critical illness in children [10], *β* values on the different time points were selected for further analyses. These 159 CpG-sites became differentially methylated in leukocytes of critically ill children by PICU discharge (*n* = 814) as compared with healthy matched peers, but were not yet differentially methylated upon PICU admission. CpG-sites that were already differentially methylated upon PICU admission had been excluded preceding this analysis to avoid detection of changes explained by premorbid conditions or illness-induced alterations in leukocyte composition. Early-PN, independent of its slowing effect on recovery, contributed to differential methylation of 37 of the 159 CpG-sites [10].

For the current time course study, of the 159 previously identified CpG-sites of which methylation was shown to be altered by critical illness and the 37 previously shown to be affected by the use of early-PN versus late-PN, only those with normal methylation upon PICU admission in the current subset were selected for further analysis [10].

### Statistical analyses

In a first part of this study, we focused on a *statistical* approach to identify differences in DNA methylation over time in PICU patients versus healthy children with the use of ANOVA and application of a false discovery rate (FDR) of 0.05 to account for multiple testing. CpG-sites already differentially methylated at PICU admission in the subset selected for the present study were first identified, revealing 12 CpG-sites to be discarded for further time profile analyses (Additional file 8). Following a similar approach, we then investigated whether the 147 remaining CpG-sites were already differentially methylated at three earlier time points (PICU day 3, 5 and 7) preceding PICU discharge.

Next, we applied a *descriptive* approach to study the altered methylation of each of these CpG-sites separately via a within-patient time course pattern recognition analysis. To this end, we plotted the DNA methylation changes (∆ DNA methylation) from admission to the respective time points (day 3, day 5, day 7 and last day in PICU) and searched for patterns among these time series. We first identified pattern categories based on the early response, i.e. change in DNA methylation status between admission and day 3, taking into account the *p* value of paired *t* tests and direction of change. Given the specific descriptive aim of pattern recognition, no correction was done for multiple comparisons. These pattern categories were subsequently further categorised for the visualised time course beyond day 3. To reduce bias induced by length of stay, we performed sensitivity analyses for the patients who stayed in PICU for at least 3 days, at least 5 days or at least 7 days, respectively.

Finally, we followed a similar approach to study the impact of early-PN versus late-PN on the time course of the CpG-sites affected by this intervention. Changes in DNA methylation status in early-PN and late-PN patients were compared with Student *t* test. Again, given the specific aim of comparing patterns, no correction for multiple comparisons was done.

Analyses were performed with the use of Partek Genomics Suite® (Partek, St. Louis, MO), R version 3.5.3 and JMP© version 14.0.0 (SAS Institute, Inc, Cary, NC). *p* values ≤ 0.05 or FDR cut-off of 0.05 was considered to indicate statistical significance.

## Supplementary information

**Additional file 1.** Definition of educational and occupational level of parents

**Additional file 2.** Definition of ‘syndrome’.

**Additional file 3.** Total energy intake of early-PN and late-PN patients on the first 7 days in PICU. Bar graphs showing total energy intake of early-PN and late-PN patients on the first 7 days in PICU.

**Additional file 4.** Location and gene-related protein functions of 147 studied CpG-sites. Location of the 147 studied CpG-sites within gene section or intergenic region and corresponding protein function if applicable, ordered according to the different patterns identified.

**Additional file 5.** Summary of ANOVA results comparing DNA methylation levels in patients at different time points during PICU stay versus healthy children. DNA methylation levels of the 147 CpG-sites that were not yet differentially methylated in patients upon PICU admission versus healthy children were compared for patients at day 3, day 5 and day 7 in PICU versus healthy children with the use of ANOVA and application of a false discovery rate of 0.05, with reporting of unadjusted *p* values.

**Additional file 6.** Evolution of DNA methylation over time of pattern 1–4 in individual critically ill patients. Detailed time profiles are shown for ten patients of representative CpG-sites classified to pattern 1, pattern 2, pattern 3, and pattern 4.

**Additional file 7.** Heatmaps of changes in methylation status of all studied CpG-sites of patterns 1–4. Heatmaps summarising the changes in methylation status of the CpG-sites classified to pattern 1, pattern 2, pattern 3 and pattern 4.

**Additional file 8.** Identification of CpG-sites differentially methylated in patients upon PICU admission as compared with healthy children. DNA methylation levels of patients upon PICU admission were compared with those of healthy children with the use of ANOVA and application of a false discovery rate of 0.05. Unadjusted *p* values for the 12 CpG-sites already differentially methylated in patients upon PICU admission versus healthy controls are shown.

## Data Availability

Data sharing will be considered only on a collaborative basis with the principal investigators, after evaluation of the proposed study protocol and statistical analysis plan.

## Abbreviations

PEPaNIC: Paediatric Early versus Late Parenteral Nutrition in Intensive Care Unit
PICU: Paediatric intensive care unit
PN: Parenteral nutrition
PeLOD: Paediatric logistic organ dysfunction
SEM: Standard error of the mean
STRONGkids: Screening tool for risk on nutritional status and growth
PIM3: Paediatric index of mortality 3
