# Supplementary material for: Time course of altered DNA methylation evoked by critical illness and by early administration of parenteral nutrition in the paediatric ICU
Source: Clin Epigenetics. 2020 Oct 20;12:155. doi: 10.1186/s13148-020-00947-w (PMC7576729; doi:10.1186/s13148-020-00947-w)
Supplement: Supplementary file 1 — Additional file 1. Definition of educational and occupational level of parents [file 13148_2020_947_MOESM1_ESM.docx]

**Additional file 1. Definition of educational and occupational level of parents**

**Educational level of parents**

The educational level is calculated based upon the 3-point scale subdivision as made by the Algemene Directie Statistiek (Belgium; statbel.fgov.be/nl/) and the Centraal Bureau voor de Statistiek (The Netherlands; statline.cbs.nl): low (1), middle (2) and high (3) educational level. The average score of the paternal and maternal educational level was calculated.

**Occupational level of parents**

The occupational level is calculated based upon the international Isco System 4-point scale for professions (https://www.ilo.org/public/english/bureau/stat/isco/isco08/index.htm). In case one of the parents filled in two jobs in the questionnaire, the highest Isco code level was used. In case ‘unemployed’, ‘disabled’, ‘student’, or ‘housewife/houseman’ was filled in, an Isco code level of 1 was given to that parent. When the parents described their profession as ‘employee’, ‘worker’, ‘liberal profession’, or ‘retired’, they were given an Isco code level of 2. The average score of the paternal and maternal occupational level was calculated.
