# Supplementary material for: Time course of altered DNA methylation evoked by critical illness and by early administration of parenteral nutrition in the paediatric ICU
Source: Clin Epigenetics. 2020 Oct 20;12:155. doi: 10.1186/s13148-020-00947-w (PMC7576729; doi:10.1186/s13148-020-00947-w)
Supplement: Supplementary file 2 — Additional file 2. Definition of ‘syndrome’. [file 13148_2020_947_MOESM2_ESM.docx]

**Additional file 2**. **Definition of ‘syndrome’**

A prerandomisation syndrome or illness *a priori* defined as affecting or possibly affecting neurocognitive development, and which is subdivided in the following categories:

- Genetically confirmed syndrome or pathogenic chromosomal abnormality
- Clearly defined syndrome, association or malformation without (identified) genetic aberration
- Polymalformative syndrome of unknown aetiology
- Clear auditory or visual impairment without specified syndrome
- Congenital hypothyroidism due to thyroid agenesis
- Brain tumour or tumour with intracranial metastatic disease
- Paedopsychiatric disorder (e.g. autism spectrum disorder, (treatment for) attention deficit hyperactivity disorder)
- Severe medical disorder, not primarily neurologic, but suspected to alter psychomotor and/or mental performance
- Severe neonatal problem (e.g. severe asphyxia)
- Severe craniocerebral trauma or near-drowning
- Severe infectious encephalitis or drug-induced encephalopathy
- Infectious meningitis, encephalitis or Guillain-Barré
- Resuscitation and/or need for extracorporeal membrane oxygenation prior to randomisation
- Severe convulsions or stroke prior to randomisation
