# Supplementary material for: Time course of altered DNA methylation evoked by critical illness and by early administration of parenteral nutrition in the paediatric ICU
Source: Clin Epigenetics. 2020 Oct 20;12:155. doi: 10.1186/s13148-020-00947-w (PMC7576729; doi:10.1186/s13148-020-00947-w)
Supplement: Supplementary file 3 — Additional file 3. Total energy intake of early-PN and late-PN patients on the first 7 days in PICU. Bar graphs showing total energy intake of early-PN and late-PN patients on the first 7 days in PICU. [file 13148_2020_947_MOESM3_ESM.docx]

**Additional file 3. Total energy intake of early-PN and late-PN patients on the first 7 days in PICU**

**
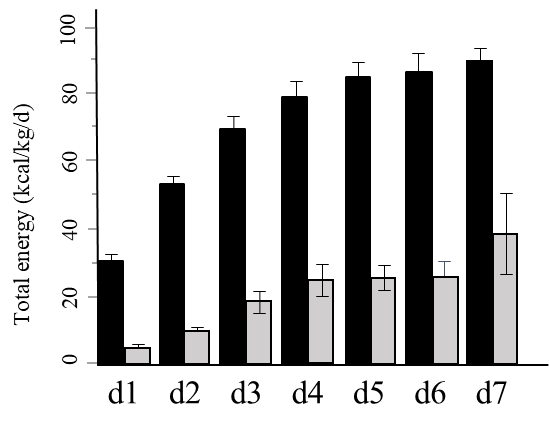
**

Black bars represent the daily total amount of energy (kilocalories per kg per day) for early-PN patients and grey bars for late-PN patients. Data are presented as means and standard errors.
