# Supplementary material for: Time course of altered DNA methylation evoked by critical illness and by early administration of parenteral nutrition in the paediatric ICU
Source: Clin Epigenetics. 2020 Oct 20;12:155. doi: 10.1186/s13148-020-00947-w (PMC7576729; doi:10.1186/s13148-020-00947-w)
Supplement: Supplementary file 4 — Additional file 4. Location and gene-related protein functions of 147 studied CpG-sites. Location of the 147 studied CpG-sites within gene section or intergenic region and corresponding protein function if applicable, ordered according to the different patterns identified. [file 13148_2020_947_MOESM4_ESM.docx]

**Additional file 4. Location and gene-related protein functions of 147 studied CpG-sites**

| **CpG-site** | **Gene section** | **Gene symbol** | **Gene-related protein functions** | **Summary functions** | **Time course pattern** |
| --- | --- | --- | --- | --- | --- |
| cg00490406 | Promoter | AIM2 | innate immune response; neuronal morphology regulation; behavior, auditory fear memory and locomotor activity; neurological/psychiatric diseases | Cerebral/Neuronal | 1 |
| cg00771778 | 5' UTR/ Non-coding | LYST | sorting endosomal resident proteins into late multivesicular endosomes; cognitive decline, parkinsonism, cerebellar deficits, spasticity and peripheral neuropathy in Chediak-Higashi syndrome | Cerebral/Neuronal | 1 |
| cg02244028 | 5' UTR | SCN11A | action potential initiation and propagation in excitable cells, including nerve, muscle, and neuroendocrine cells; mediating brain-derived neurotrophic factor-evoked membrane depolarization; major effector of peripheral inflammatory pain hypersensitivity | Peripheral neuronal | 1 |
| cg02293222 | Intergenic |  |  |  | 1 |
| cg03083216 | Intron | XRCC4 | DNA repair; physical development; central nervous system development | Cerebral/Neuronal | 1 |
| cg03417712 | Intron | SARDH | sarcosine degradation | Metabolism | 1 |
| cg03778523 | Intergenic |  |  |  | 1 |
| cg04588138 | Intergenic |  |  |  | 1 |
| cg04689867 | Promoter | ADGRE1 | cell adhesion; cell-cell interactions in the immune system | Inflammation/Immune system | 1 |
| cg05112114 | Intron/ Non-coding | INO80 | transcriptional regulation; DNA replication and repair; microcephaly; developmental delay; weight regulation | Cerebral/Neuronal | 1 |
| cg06826494 | Exon | SVIL | myogenesis (myogenic membrane structure and differentiation); cell migration | Growth/Development/Locomotion | 1 |
| cg07078732 | Intergenic |  |  |  | 1 |
| cg08097676 | Intron | METTL16 | regulating S-adenosyl-methionine homeostasis | Gene expression/DNA regulation/Epigenetic regulation | 1 |
| cg09366519 | 5' UTR/ Promoter | HSD11B1 | cortisol metabolism; verbal learning and recall, concentration, and memory, depression, dementia, and delirium | Cerebral/Neuronal | 1 |
| cg10207609 | 5' UTR/ Promoter | CD36 | regulating many signaling processes; fatty acid transport; brain lipid sensing and nervous control of energy balance; behavior, spatial learning and memory; neuropsychiatric and neurodegenerative disorders | Cerebral/Neuronal | 1 |
| cg10403028 | Non-coding | TEX41 | non-coding RNA | Non-coding RNA/Pseudogene | 1 |
| cg10480329 | Intron | CENPA | propagating centromere identity through replication and cell division; concentration, psychomotor speed; verbal memory | Cerebral/Neuronal | 1 |
| cg11047783 | Intron | KAT6B | transcriptional regulation; cerebral cortex development; cognition; ADHD; intellectual disability; microcephaly and growth regulation | Cerebral/Neuronal | 1 |
| cg11180972 | Intron | CHST11 | sulfating chondroitin; skeletal development; brain development; locomotion; ADHD | Cerebral/Neuronal | 1 |
| cg14800111 | Intergenic |  |  |  | 1 |
| cg17533201 | Intron | RAB11FIP4 | regulating endocytic traffic; cytokinesis | Intra-intercellular Signaling/Transport | 1 |
| cg18685299 | Intergenic |  |  |  | 1 |
| cg19746667 | Intergenic |  |  |  | 1 |
| cg20093635 | Intron | COL15A1 | stabilizing microvessels and muscle cells; peripheral nerve maturation; sensory nerve conduction; neuromuscular development | Peripheral neuronal | 1 |
| cg20132375 | Intergenic |  |  |  | 1 |
| cg20700099 | Intron/ Non-coding | DPP8 | T-cell activation and immune function (possibly, based on similarities with DPP4); ischemia-triggered inflammation and neurodegeneration | Cerebral/Neuronal | 1 |
| cg20744727 | Promoter | CD93 | intercellular adhesion and clearance of apoptotic cells; phagocytic capacity of activated microglia, associated with neurodegeneration | Cerebral/Neuronal | 1 |
| cg20944115 | Intergenic |  |  |  | 1 |
| cg22076676 | Intron/ Non-coding | THADA | apoptosis; adaptive thermogenesis; homeostasis; neuroinflammation and multiple sclerosis | Cerebral/Neuronal | 1 |
| cg22979802 | Intron | CLNK | regulating immunoreceptor signaling | Inflammation/Immune system | 1 |
| cg23057326 | 5' UTR | HIVEP3 | transcriptional regulation; cell progression and differentiation; regulating bone formation; cognitive function; pain modulation | Cerebral/Neuronal | 1 |
| cg23104428 | Intron | TTC7A | regulation of phosphatidylinositol 4-phosphate synthesis; targeting PI4kinase to plasmamembrane; intestinal development | Intra-intercellular Signaling/Transport | 1 |
| cg23391288 | Intergenic |  |  |  | 1 |
| cg24533408 | Intergenic |  |  |  | 1 |
| cg24785495 | Intergenic |  |  |  | 1 |
| cg24954967 | Intergenic |  |  |  | 1 |
| cg25699964 | Intergenic |  |  |  | 1 |
| cg26082814 | 5' UTR | LOC101928841 | unknown | Non-coding RNA/Pseudogene | 1 |
| cg26685998 | Intergenic |  |  |  | 1 |
| cg00025197 | Intergenic |  |  |  | 1 |
| cg00995147 | Intergenic |  |  |  | 1 |
| cg01095594 | Non-coding | LOC101927043 | non-coding RNA | Non-coding RNA/Pseudogene | 1 |
| cg01860912 | Intron | IL19 | transcriptional activation by activating STAT3; inflammatory responses; microglia activation; Alzheimer's disease | Cerebral/Neuronal | 1 |
| cg04837642 | Intergenic |  |  |  | 1 |
| cg05371584 | Intron/ Non-coding | TBC1D8 | unknown (putative role in GTPase-activation of Rab family proteins) | Intra-intercellular Signaling/Transport | 1 |
| cg05706766 | Intergenic |  |  |  | 1 |
| cg05889889 | Intron | RTTN | axial rotation, left-right organ specification; differentiation of the neural tube, and looping of the heart tube during development; maintenance of normal ciliary structure | Cerebral/Neuronal | 1 |
| cg06297318 | Intergenic |  |  |  | 1 |
| cg06329036 | Intron | SORCS2 | expression of BDNF (brain-derived neurotrophic factor, regulator of structural, synaptic and morphological plasticity of the brain); high expression in central neurvous system; bipolar disorder, schizophrenia; Alzheimer’s disease | Cerebral/Neuronal | 1 |
| cg06449934 | 5' UTR | GPER1 | numerous intracellular signaling pathways among the cardiovascular, endocrine, reproductive, immune and central nervous systems; regulation of hippocampal memory and cognition; social and spatial recognition learning; working memory; synaptic transmission; anxiety | Cerebral/Neuronal | 1 |
| cg07170641 | 5' UTR/ Intron | MS4A6A | signal transduction in hematopoietic cells and in the brain | Cerebral/Neuronal | 1 |
| cg08442088 | 5' UTR/ Intron | DBNL | reorganization of the actin cytoskeleton; formation of cell projections, such as neurites; morphogenesis of neurons and synapse formation | Cerebral/Neuronal | 1 |
| cg08965078 | Intergenic |  |  |  | 1 |
| cg09194657 | Intron/ Non-coding | TUBGCP2 | microtubule nucleation at the centrosome | Gene expression/DNA regulation/Epigenetic regulation | 1 |
| cg10862350 | Intron | PDE4B | signal transduction processes a.o. in central nervous system; schizophrenia; bipolar disorder | Cerebral/Neuronal | 1 |
| cg11320225 | Intergenic |  |  |  | 1 |
| cg13140887 | Intergenic |  |  |  | 1 |
| cg14116399 | Intergenic |  |  |  | 1 |
| cg14172797 | Intron | PRKCA | many different cellular processes, such as cell proliferation, differentiation and apoptosis, cell cycle checkpoint, and cell volume control; cancer development; episodic remembering (memory); mood regulation; behavior; choroid gliomas; highly expressed in brain | Cerebral/Neuronal | 1 |
| cg14450616 | 5' UTR | PLD3 | hydrolysis of membrane phospholipids and processing of amyloid-beta precursor protein; neuronal development and survival, neurotransmission; visual learning, memory, speed and flexibility; Alzheimer’s disease; highly expressed in the brain | Cerebral/Neuronal | 1 |
| cg14748515 | Intergenic |  |  |  | 1 |
| cg15209896 | 5' UTR | STPG1 | apoptosis | Oncogenic/apoptosis | 1 |
| cg15296538 | Non-coding | AACSP1 | pseudogene | Non-coding RNA/Pseudogene | 1 |
| cg15453708 | 5' UTR/ Intron | PRKAG2 | regulating cellular energy metabolism including inhibition of protein, carbohydrate and lipid biosynthesis, and cell growth and proliferation | Metabolism | 1 |
| cg18012268 | Intergenic |  |  |  | 1 |
| cg18978661 | Non-coding | LINC01599 | non-coding RNA | Non-coding RNA/Pseudogene | 1 |
| cg19323261 | Intergenic |  |  |  | 1 |
| cg22180675 | 3' UTR/ Intron | MIR486-1 | miRNA; skeletal muscle development | Growth/Development/Locomotion | 1 |
| cg22535104 | Intron | HIP1 | clathrin-mediated endocytosis and trafficking; regulating AMPA receptor trafficking in the central nervous system; regulating presynaptic nerve terminal activity | Cerebral/Neuronal | 1 |
| cg23668476 | Intron | TPST1 | catalyzing the O-sulfation of tyrosine residues of polypeptides | Metabolism | 1 |
| cg23714751 | Intron | MKL1 | regulating smooth muscle cell differentiation; brain development; neuronal survival; Alzheimer’s disease; schizophrenia; skeletal myogenic differentiation; growth | Cerebral/Neuronal | 1 |
| cg24475272 | 3' UTR/ Intron | SETD7 | histone methylation, with impact on transcriptional activation of genes such as collagenase or insulin | Gene expression/DNA regulation/Epigenetic regulation | 1 |
| cg25026693 | Intron | UBASH3B | promoting accumulation of activated target receptors, such as T-cell receptors and EGFR, on the cell surface | Intra-intercellular Signaling/Transport | 1 |
| cg25300481 | Intergenic |  |  |  | 1 |
| cg25808826 | Intergenic |  |  |  | 1 |
| cg26308668 | Intron | SRGAP1 | regulating neuronal development and migration; mental retardation; autism; schizophrenia | Cerebral/Neuronal | 1 |
| cg00101629 | Intron | KAZN | desmosome assembly, cell adhesion, cytoskeletal organization, and epidermal differentiation | Cell structure | 2 |
| cg00182635 | Intergenic |  |  |  | 2 |
| cg00687889 | Intergenic |  |  |  | 2 |
| cg01842756 | Intron/ Non-coding | RNF217 | apoptosis signaling | Oncogenic/apoptosis | 2 |
| cg01858828 | Intergenic |  |  |  | 2 |
| cg01912915 | Intergenic |  |  |  | 2 |
| cg02435538 | Promoter | RHBDD2 | unknown (putative involvement in intramembrane proteolysis); highly expressed in brain | Cerebral/Neuronal | 2 |
| cg02918489 | Intergenic |  |  |  | 2 |
| cg03116607 | Intergenic |  |  |  | 2 |
| cg04193065 | Intergenic |  |  |  | 2 |
| cg05038391 | Intergenic |  |  |  | 2 |
| cg06636137 | Intron | PHF10 | proliferation of neural progenitors; dendrite growth | Cerebral/Neuronal | 2 |
| cg08285151 | Intron/ 5'UTR/ Promoter | HDAC9 | histone deacetylation; inhibiting skeletal myogenesis; involved in heart development; protecting neurons from apoptosis | Cerebral/Neuronal | 2 |
| cg08556511 | Intron | YAP1 | regulation of the Hippo signaling pathway which is involved in development, growth, repair and homeostasis; progression of multiple cancers, including glioblastoma; brain development; Huntington disease | Cerebral/Neuronal | 2 |
| cg08948258 | Intergenic |  |  |  | 2 |
| cg10422093 | Intergenic |  |  |  | 2 |
| cg10507267 | Intergenic |  |  |  | 2 |
| cg10732094 | Intergenic |  |  |  | 2 |
| cg10866825 | Intergenic |  |  |  | 2 |
| cg11520439 | 3' UTR/ Non-coding | NIPSNAP3B | vesicular trafficking | Intra-intercellular Signaling/Transport | 2 |
| cg12274883 | Intergenic |  |  |  | 2 |
| cg12928479 | Exon | NLRC5 | regulating the NF-kappa-B and type I interferon signaling pathways; immunity; neuroimmune and neuroinflammatory processes | Cerebral/Neuronal | 2 |
| cg14109551 | Intron | CEP85L | brain tumors; attention deficit hyperactivity disorder (ADHD); bipolar disorders | Cerebral/Neuronal | 2 |
| cg15809077 | Intergenic |  |  |  | 2 |
| cg16029189 | Intergenic |  |  |  | 2 |
| cg16301196 | 3' UTR | PLA2G15 | hydrolyzing lysophosphatidylcholine to glycerophosphorylcholine and a free fatty acid; phospholipid degradation | Metabolism | 2 |
| cg16651946 | Intron | UBP1 | transcriptional activation | Gene expression/DNA regulation/Epigenetic regulation | 2 |
| cg17522929 | 5' UTR/ Intron | PKM | glycolysis; transcriptional activation; caspase-independent cell death of tumor cells; may mediate metabolic effects of thyroid hormone; involved in bacterial pathogenesis (adherence of bacteria to human cells); brain development; neuronal differentiation | Cerebral/Neuronal | 2 |
| cg17636223 | Intergenic |  |  |  | 2 |
| cg21329012 | Intron | MAP3K2 | MAP kinase and NF-kappaB signaling pathways; BDNF functioning in central nervous system neurons; bone formation; myogenesis | Cerebral/Neuronal | 2 |
| cg22645359 | 3' UTR/ Intron | TCF7L2 | transcriptional regulation in the Wnt signaling pathway; blood glucose homeostasis; related to neurodevelopment and plasticity of mature neurons; memory, visual motor/fine motor function, emotional functioning, behavior and speech; schizophrenia; ADHD | Cerebral/Neuronal | 2 |
| cg24451839 | Intergenic |  |  |  | 2 |
| cg25551043 | Intron | MYOF | plasmalemma repair mechanism of endothelial cells that permits rapid resealing of membranes disrupted by mechanical stress; endocytic recycling; Duchenne muscular dystrophy | Growth/Development/Locomotion | 2 |
| cg26179530 | Intron | AAK1 | regulating clathrin-mediated endocytosis; transcriptional activation; neurotrophic factor signaling and dendrite growth regulation; cognitive functioning/learning and memory; neurodegenerative disorders; schizophrenia | Cerebral/Neuronal | 2 |
| cg27229520 | Intergenic |  |  |  | 2 |
| cg27518631 | Intergenic |  |  |  | 2 |
| cg05174290 | Intron/ Non-coding | ATAD2B | chromatin-related function; neuronal differentiation; tumor progression | Cerebral/Neuronal | 3 |
| cg05304729 | Promoter | MNDA | transcriptional regulation in the myeloid lineage | Gene expression/DNA regulation/Epigenetic regulation | 3 |
| cg03958078 | Intron | IGFBP7 | regulating IGF availability; cell growth regulation; senescence; apoptosis; cancer; memory consolidation; neurogenesis | Cerebral/Neuronal | 3 |
| cg06572103 | 5' UTR/ Intron | TNFSF11 | osteoclast differentiation and activation (and bone loss); T-cell proliferation and T-cell-dependent immune respons; apoptosis; skeletal pathologies: osteogenesis imperfecta, osteolysis in diabetic neuropathy, bone cancer pain, Charchot Marie Tooth disease and other metabolic bone disorders | Growth/Development/Locomotion | 3 |
| cg06827976 | 5' UTR | FGR | regulating immune responses; cell migration; cytoskeleton reorganization | Inflammation/Immune system | 3 |
| cg09021674 | Intron | EIF3E | initiation of protein synthesis; growth; intellectual ability; autism-spectrum and neurodegenerative disorders | Cerebral/Neuronal | 3 |
| cg09396032 | 5' UTR/ Intron | GSE1 | oncogene | Oncogenic/apoptosis | 3 |
| cg10840227 | Intron | TPP2 | proteolysis; removing tripeptides from longer peptides | Metabolism | 3 |
| cg11919725 | Intergenic |  |  |  | 3 |
| cg26760894 | Non-coding | TRAF3IP2-AS1 | long non-coding RNA; cocaine abuse; bipolar disease | Cerebral/Neuronal | 3 |
| cg00507757 | Intron | CS | catalyzing the synthesis of citrate from oxaloacetate and acetyl coenzyme A; neurological/ neurodegenerative diseases; autism spectrum and bipolar disorders; social function and behavior | Cerebral/Neuronal | 4 |
| cg01891736 | 5' UTR/ Non-coding | LITAF | regulating TNF-alpha expression; endosomal protein trafficking, targeting proteins for lysosomal degradation; Charcot Marie Tooth disease type 1C peripheral neuropathy | Peripheral neuronal | 4 |
| cg06975311 | Promoter | SIPA1 | regulating the Ras signaling pathway; cell cycle progression | Intra-intercellular Signaling/Transport | 4 |
| cg07375256 | Intron | ZSCAN25 | transcriptional regulation (DNA binding and protein-protein interactions); genetic variation in ZSCAN25 has been associated with body weight, hip and brachial circumference. | Growth/Development/Locomotion | 4 |
| cg08822136 | Intergenic |  |  |  | 4 |
| cg10170678 | Intron | RIN3 | Ras signaling pathway; neuronal signaling; Alzheimer’s disease | Cerebral/Neuronal | 4 |
| cg10322118 | Non-coding | B4GALT1-AS1 | non-coding RNA | Non-coding RNA/Pseudogene | 4 |
| cg14071298 | Intergenic |  |  |  | 4 |
| cg14141074 | Promoter/ Intron | TBC1D1 | cell cycle and differentiation of various tissues; insulin-stimulated glucose uptake into cells (trafficking of GLUT-4) | Intra-intercellular Signaling/Transport | 4 |
| cg14364797 | 3' UTR | FNBP1 | regulation of the actin cytoskeleton; spine formation/neurite branching; neuronal network formation; information processing | Cerebral/Neuronal | 4 |
| cg16513984 | Intron | TNFRSF1B | regulating anti-apoptotic pathways; protecting neurons from apoptosis by stimulating anti-oxidative pathways | Cerebral/Neuronal | 4 |
| cg17022038 | Exon | MAST3 | unknown; microtubule-associated serine/threonine-protein kinase gene-3 | Metabolism | 4 |
| cg17804886 | 5' UTR/ Intron | RASA3 | negatively regulating the Ras signaling pathway | Intra-intercellular Signaling/Transport | 4 |
| cg21695395 | Intron | ETS1 | transcriptional regulation of numerous genes; stem cell development; cell senescence and death; tumorigenesis; angiogenesis | Gene expression/DNA regulation/Epigenetic regulation | 4 |
| cg23053742 | Non-coding | LACTB2-AS1 | non-coding RNA | Non-coding RNA/Pseudogene | 4 |
| cg23084667 | 5' UTR | GRAP2 | leukocyte-specific protein-tyrosine kinase signaling; RET signaling which is involved in brain development and maturation of dopaminergic neurons | Cerebral/Neuronal | 4 |
| cg24874433 | Non-coding | LINC00854 | non-coding RNA | Non-coding RNA/Pseudogene | 4 |
| cg26683792 | Intron | SLC35E1 | unknown (putative transporter) | Intra-intercellular Signaling/Transport | 4 |
| cg27215601 | Intron/ Non-coding | NSMCE2 | nuclear transport, transcription, chromosome segregation and DNA repair; key role in genome maintenance, suppression of mitotic recombination; dwarfism; bipolar disorder | Cerebral/Neuronal | 4 |
| cg15507942 | Promoter/5' UTR | CD36 | regulating many signaling processes; fatty acid transport; brain lipid sensing and nervous control of energy balance; behavior, spatial learning and memory; neuropsychiatric and neurodegenerative disorders | Cerebral/Neuronal | 5 |
| cg17134427 | Exon/Non-coding | ZFR | RNA binding protein involved in nucleocytoplasmic shuttling in neurons; post-implantation and gastrulation stages of development; pancreatic cancer; highly expressed in brain | Cerebral/Neuronal | 5 |
| cg21336878 | 5' UTR | LYAR | ribosomal RNA processing; transcription regulation | Gene expression/DNA regulation/Epigenetic regulation | 5 |
| cg05923857 | Exon | TCF7L2 | transcriptional regulation in the Wnt signaling pathway; blood glucose homeostasis; neurodevelopment and plasticity of mature neurons; memory, visual-motor/fine-motor function; spatial learning, emotional functioning; behavior; speech; schizophrenia; ADHD; anxiety and bipolar disorders | Cerebral/Neuronal | 6 |
| cg06637027 | Promoter | CD86 | T-lymphocyte proliferation and interleukin-2 production; microglia activation; cognitive/ neurodegenerative disturbances | Cerebral/Neuronal | 6 |
| cg07584558 | 3' UTR/ Non-coding | DEDD2 | receptor-induced apoptosis | Oncogenic/apoptosis | 6 |

Adapted supplementeray table S2 from Guïza et al [1]. Source of gene-related protein function: Entrez Gene Summary at Pubmed.com and UniProt database (www.uniprot.org), complemented with updated literature searches for links with brain and development. CpG-sites marked in gray are those of which the methylation status was significantly affected by early-PN versus late-PN. Abbreviations: AACSP1, Acetoacetyl-CoA Synthetase Pseudogene 1; AAK1, Adaptor-related protein complex 2 Associated Kinase 1; ADGRE1, Adhesion G Protein-Coupled Receptor E1; ADHD: attention deficit hyperactivity disorder; AIM2, Absent In Melanoma 2; ATAD2B, ATPase Family AAA Domain Containing 2B; B4GALT1-AS1, Beta-1,4-galactosyltransferase 1 Antisense ribonucleic acid 1; CD36, Cluster of Differentiation 36; CD86, Cluster of Differentiation 86; CD93, Cluster of Differentiation 93; CENPA, Centromere protein A; CEP85L, Centrosomal Protein 85 Like; CHST11, Carbohydrate Sulfotransferase 11; CLNK, Cytokine Dependent Hematopoietic Cell Linker; COL15A1, Collagen Type XV Alpha 1 Chain; DEDD2, Death Effector Domain Containing 2; DPP8, Dipeptidyl Peptidase 8; EIF3E, Eukaryotic Translation Initiation Factor 3 Subunit E; ETS1, E26 Transformation-Specific Proto-Oncogene 1; FGR, Feline Gardner-Rasheed Sarcoma Proto-Oncogene; FNBP1, Formin Binding Protein 1; GPER1, G Protein-Coupled Oestrogen Receptor 1; GRAP2, Growth factor Receptor-bound Protein 2-Related Adaptor Protein 2; GSE1, Genetic Suppressor Element 1; HDAC9, Histone Deacetylase 9; HIP1, Huntingtin Interacting Protein 1; HIVEP3, Human Immunodeficiency Virus Type I enhancer binding protein 3; HSD11B1, 11-Beta-Hydroxysteroid Dehydrogenase 1; IGFBP7, Insulin-like Growth Factor Binding Protein 7; IL19, Interleukin 19; INO80, inositol auxotroph 80; KAT6B, Lysine Acetyltransferase 6B; KAZN, Kazrin; LACTB2-AS1, Lactamase Beta 2 Antisense ribonucleic acid 1; LINC00854, Long Intergenic Non-Protein Coding RNA 854; LINC01599, Long Intergenic Non-Protein Coding RNA 1599; LITAF, Lipopolysaccharide Induced TNF Factor; LOC101927043, Collagen Alpha-1(II) Chain-Like 101927043; LOC101928841, Collagen Alpha-1(II) Chain-Like 101928841; LYAR, Ly1 Antibody Reactive; LYST, Lysosomal Trafficking Regulator; MAP3K2, Mitogen-Activated Protein 3 Kinase 2; MAST3, Microtubule Associated Serine/Threonine Kinase 3; METTL16, Methyltransferase Like 16; MIR486-1, MicroRNA 486-1; MKL1, Megakaryoblastic Leukaemia (Translocation) 1; MNDA, Myeloid Cell Nuclear Differentiation Antigen; MS4A6A, Membrane Spanning 4-Domains A6A; MYOF, Myoferlin; NIPSNAP3B, Nipsnap Homolog 3B; NLRC5, NLR Family CARD Domain Containing 5; NSMCE2, Non-SMC Element 2 Methyl Methanesulfonate Sensitivity Gene 21 Homolog; PDE4B, Phosphodiesterase 4B; PHF10, PHD Finger Protein 10; PICU, pediatric intensive care unit; PKM, Pyruvate Kinase Muscle; PLA2G15, Phospholipase A2 Group XV; PLD3, Phospholipase D Family Member 3; PRKAG2, Protein Kinase AMP-Activated Non-Catalytic Subunit Gamma 2; PRKCA, Protein Kinase C Alpha; RAB11FIP4, RAB11 Family Interacting Protein 4; RASA3, RAS P21 Protein Activator 3; RHBDD2, Rhomboid Domain Containing 2; RIN3, Ras And Rab Interactor 3; RNF217, Ring Finger Protein 217; RTTN, Rotatin; SARDH, Sarcosine Dehydrogenase; SCN11A, Sodium Voltage-Gated Channel Alpha Subunit 11; SETD7, SET Domain Containing Lysine Methyltransferase 7; SIPA1, Signal-Induced Proliferation-Associated 1; SLC35E1, Solute Carrier Family 35 Member E1; SORCS2, Sortilin Related VPS10 Domain Containing Receptor 2; SRGAP1, SLIT-ROBO Rho GTPase Activating Protein 1; STPG1, Sperm Tail PG-Rich Repeat Containing 1; SVIL, Supervillin; TBC1D1, TBC1 Domain Family Member 1; TBC1D8, TBC1 Domain Family Member 8; TCF7L2, Transcription Factor 7 Like 2; TEX41, Testis Expressed 41; THADA, Thyroid Adenoma-Associated Protein; TNFRSF1B, TNF Receptor Superfamily Member 1B; TNFSF11, TNF Superfamily Member 11; TPP2, Tripeptidyl Peptidase 2; TPST1, Tyrosylprotein Sulfotransferase 1; TRAF3IP2-AS1, TRAF3IP2 Antisense RNA 1; TTC7A, Tetratricopeptide Repeat Domain 7A; TUBGCP2, Tubulin Gamma Complex Associated Protein 2; UBASH3B, Ubiquitin Associated And SH3 Domain Containing B; UBP1, Upstream Binding Protein 1; XRCC4, X-Ray Repair Cross Complementing 4; YAP1, Yes Associated Protein 1; ZFR, Zinc Finger Ribonucleic acid binding protein; ZSCAN25, Zinc Finger And SCAN Domain Containing 25.

**REFERENCES**

1. Guïza F, Vanhorebeek I, Verstraete S, Verlinden I, Derese I, Ingels C, et al. Effect of early parenteral nutrition during paediatric critical illness on DNA methylation as a potential mediator of impaired neurocognitive developement: A pre-planned secondary analysis of the PEPaNIC international, randomised controlled trial. Lancet Respir Med. 2020;8:288-303
