# Supplementary material for: Time course of altered DNA methylation evoked by critical illness and by early administration of parenteral nutrition in the paediatric ICU
Source: Clin Epigenetics. 2020 Oct 20;12:155. doi: 10.1186/s13148-020-00947-w (PMC7576729; doi:10.1186/s13148-020-00947-w)
Supplement: Supplementary file 5 — Additional file 5. Summary of ANOVA results comparing DNA methylation levels in patients at different time points during PICU stay versus healthy children. DNA methylation levels of the 147 CpG-sites that were not yet differentially methylated in patients upon PICU admission versus healthy children were compared for patients at day 3, day 5 and day 7 in PICU versus healthy children with the use of ANOVA and application of a false discovery rate of 0.05, with reporting of unadjusted p values. [file 13148_2020_947_MOESM5_ESM.docx]

**Additional file 5. Summary of ANOVA results comparing DNA-methylation levels in patients at different time points during PICU stay versus healthy children.**

| **CpG sites** | **Patients at PICU day 3 vs healthy children**  **Unadjusted p-values** | **Patients at PICU day 5 vs healthy children**  **Unadjusted p-values** | **Patients at PICU day 7 vs healthy children**  **Unadjusted p-values** |
| --- | --- | --- | --- |
| cg00025197 | 0.0379151 | 0.0425888 | 0.0417417 |
| cg00101629 | 0.0669478 | 0.0161785 | 0.000373154 |
| cg00182635 | 0.14962 | 0.165695 | 0.00131518 |
| cg00490406 | 0.0231593 | 0.09753 | 0.358658 |
| cg00507757 | 0.316599 | 0.188686 | 0.05289 |
| cg00687889 | 0.461314 | 0.0760204 | 0.00287134 |
| cg00771778 | 0.000373688 | 0.0137246 | 0.000718558 |
| cg00995147 | 2.4291e-05 | 0.000230591 | 1.86773e-05 |
| cg01095594 | 0.000773122 | 0.000758295 | 0.00233217 |
| cg01842756 | 0.00209329 | 0.0046811 | 3.31277e-06 |
| cg01858828 | 0.0282092 | 0.0332508 | 0.00753247 |
| cg01860912 | 0.0679602 | 0.0740575 | 0.0200113 |
| cg01891736 | 0.101494 | 0.0160773 | 0.00125888 |
| cg01912915 | 0.00323391 | 0.00580904 | 0.00094633 |
| cg02244028 | 0.00436229 | 0.0538398 | 0.0428543 |
| cg02293222 | 0.0461482 | 0.128759 | 0.240834 |
| cg02435538 | 0.329788 | 0.0713565 | 0.00718316 |
| cg02918489 | 1.9114e-05 | 4.21475e-06 | 1.16421e-06 |
| cg03083216 | 0.00829376 | 0.015105 | 0.0228779 |
| cg03116607 | 0.0038922 | 0.0034537 | 7.26867e-05 |
| cg03417712 | 0.0486186 | 0.236494 | 0.103162 |
| cg03778523 | 0.0173284 | 0.00248981 | 0.000266211 |
| cg03958078 | 0.0077059 | 0.00479264 | 2.09965e-06 |
| cg04193065 | 0.00374984 | 0.00184652 | 7.32005e-05 |
| cg04588138 | 0.00617608 | 0.0269701 | 0.00168162 |
| cg04689867 | 0.0154674 | 0.000802354 | 5.6144e-05 |
| cg04837642 | 0.117317 | 0.0390917 | 0.0132213 |
| cg05038391 | 0.882396 | 0.718235 | 0.294733 |
| cg05112114 | 0.000570853 | 0.00195074 | 0.000973866 |
| cg05174290 | 0.150996 | 0.0510389 | 0.0223497 |
| cg05304729 | 5.2199e-11 | 9.96015e-13 | 8.4863e-08 |
| cg05371584 | 0.086601 | 0.0355268 | 0.000992147 |
| cg05706766 | 0.000445572 | 0.00169996 | 0.00335905 |
| cg05889889 | 0.00248108 | 0.00518645 | 0.000219994 |
| cg05923857 | 0.0147492 | 0.0238721 | 0.0105084 |
| cg06297318 | 0.0245612 | 0.00271116 | 0.00962049 |
| cg06329036 | 0.067893 | 0.00497367 | 0.00159316 |
| cg06449934 | 0.000684426 | 2.78077e-05 | 4.06543e-06 |
| cg06572103 | 9.15445e-05 | 0.000565041 | 0.00049509 |
| cg06636137 | 0.00231509 | 0.029539 | 4.67372e-06 |
| cg06637027 | 0.31151 | 0.216542 | 0.202728 |
| cg06826494 | 0.0345482 | 0.226197 | 0.0411103 |
| cg06827976 | 0.00143211 | 0.00803597 | 0.0230379 |
| cg06975311 | 0.988075 | 0.0709766 | 0.00137967 |
| cg07078732 | 0.00138563 | 0.00384264 | 0.00299359 |
| cg07170641 | 6.38812e-06 | 9.66623e-05 | 3.50855e-07 |
| cg07375256 | 0.00496311 | 0.00360402 | 0.000980335 |
| cg07584558 | 0.368309 | 2.8671e-05 | 1.0796e-07 |
| cg08097676 | 2.20087e-05 | 0.000368544 | 0.0043335 |
| cg08285151 | 0.0126967 | 0.000709138 | 0.000832509 |
| cg08442088 | 0.0261946 | 0.000952127 | 0.000517266 |
| cg08556511 | 0.00104818 | 0.000380848 | 6.69629e-08 |
| cg08822136 | 0.143419 | 0.0582631 | 0.0146469 |
| cg08948258 | 0.00324803 | 0.00257042 | 0.000368064 |
| cg08965078 | 0.00396356 | 0.0171149 | 0.0026348 |
| cg09021674 | 0.21346 | 0.142811 | 0.196578 |
| cg09194657 | 0.144284 | 0.366899 | 0.110036 |
| cg09366519 | 2.59098e-05 | 7.50664e-07 | 2.39436e-07 |
| cg09396032 | 0.0406144 | 0.0380612 | 3.81553e-06 |
| cg10170678 | 0.00700549 | 0.00515271 | 0.0022536 |
| cg10207609 | 0.0322318 | 0.030492 | 0.00168271 |
| cg10322118 | 2.47199e-05 | 6.15535e-08 | 1.13986e-05 |
| cg10403028 | 0.000852535 | 4.08565e-05 | 1.99413e-05 |
| cg10422093 | 0.15526 | 0.138496 | 0.000379429 |
| cg10480329 | 0.000270163 | 0.00474519 | 0.000293551 |
| cg10507267 | 0.000153898 | 0.00050146 | 1.51499e-05 |
| cg10732094 | 0.000106787 | 1.3723e-05 | 1.89721e-06 |
| cg10840227 | 0.00202408 | 0.00633158 | 0.0167013 |
| cg10862350 | 0.00873422 | 0.0467109 | 0.00113431 |
| cg10866825 | 0.00775227 | 0.00629129 | 0.000675251 |
| cg11047783 | 0.0652828 | 0.11544 | 0.00630572 |
| cg11180972 | 0.00565992 | 0.00996613 | 0.00928114 |
| cg11320225 | 0.0102175 | 0.0108074 | 0.000677985 |
| cg11520439 | 0.029015 | 0.00635128 | 0.000332994 |
| cg11919725 | 0.223922 | 0.623869 | 0.00646666 |
| cg12274883 | 0.0341279 | 0.032458 | 0.00549176 |
| cg12928479 | 0.432193 | 0.101534 | 0.00537885 |
| cg13140887 | 0.0250125 | 0.0709072 | 0.0164716 |
| cg14071298 | 0.158747 | 0.00723093 | 5.36208e-05 |
| cg14109551 | 0.000826598 | 5.54118e-05 | 1.56586e-06 |
| cg14116399 | 0.000188034 | 7.89685e-05 | 3.15219e-05 |
| cg14141074 | 0.0140974 | 0.000811139 | 0.000118947 |
| cg14172797 | 0.0100389 | 0.00336522 | 0.00032972 |
| cg14364797 | 0.0361255 | 0.00254535 | 0.00650097 |
| cg14450616 | 0.0363409 | 0.0196373 | 0.00129958 |
| cg14748515 | 0.04631 | 0.0658677 | 0.0017332 |
| cg14800111 | 0.000697808 | 0.000749754 | 0.000720657 |
| cg15209896 | 0.000267248 | 0.00059792 | 1.31551e-06 |
| cg15296538 | 0.00189376 | 0.00162296 | 0.000210473 |
| cg15453708 | 0.00188543 | 0.00212804 | 0.000589266 |
| cg15507942 | 0.729499 | 0.589198 | 0.302455 |
| cg15809077 | 0.0265151 | 0.00256272 | 3.9342e-05 |
| cg16029189 | 0.108517 | 0.0057257 | 0.000326157 |
| cg16301196 | 0.106216 | 0.0156611 | 2.86562e-05 |
| cg16513984 | 0.00012179 | 8.17901e-05 | 2.74324e-06 |
| cg16651946 | 0.0716869 | 0.0062802 | 0.00311072 |
| cg17022038 | 1.04805e-07 | 6.02766e-07 | 1.49086e-07 |
| cg17134427 | 0.0285962 | 0.128911 | 0.0023495 |
| cg17522929 | 0.000981499 | 0.000195982 | 3.37626e-06 |
| cg17533201 | 0.00841293 | 0.0112259 | 0.0189377 |
| cg17636223 | 0.790662 | 0.682568 | 0.211869 |
| cg17804886 | 0.12726 | 0.0143529 | 0.0319047 |
| cg18012268 | 0.00458257 | 0.00327709 | 8.58555e-06 |
| cg18685299 | 0.000622704 | 0.00437446 | 5.26226e-07 |
| cg18978661 | 0.00275421 | 0.0246203 | 0.00565932 |
| cg19323261 | 0.00616049 | 0.105476 | 0.0157214 |
| cg19746667 | 0.000367255 | 0.00280512 | 0.000324289 |
| cg20093635 | 0.000168613 | 4.76877e-06 | 1.13643e-07 |
| cg20132375 | 0.00136531 | 0.000181064 | 0.000743432 |
| cg20700099 | 0.00064246 | 0.00146292 | 0.000515166 |
| cg20744727 | 0.0342019 | 0.0412916 | 0.00855352 |
| cg20944115 | 0.0484942 | 0.0134531 | 0.0010551 |
| cg21329012 | 0.14946 | 0.0113962 | 0.000122353 |
| cg21336878 | 0.0916813 | 0.0254871 | 0.000543961 |
| cg21695395 | 0.0224891 | 0.00754018 | 2.11581e-06 |
| cg22076676 | 0.0074453 | 0.0275805 | 0.000674659 |
| cg22180675 | 0.00525237 | 0.00540774 | 0.000865027 |
| cg22535104 | 0.0647501 | 0.0201645 | 0.00937265 |
| cg22645359 | 0.388585 | 0.574175 | 0.00606025 |
| cg22979802 | 0.00104355 | 0.00152565 | 6.42041e-05 |
| cg23053742 | 0.047173 | 0.00552842 | 0.00101063 |
| cg23057326 | 0.180271 | 0.205309 | 0.483896 |
| cg23084667 | 0.467064 | 0.257266 | 0.00875052 |
| cg23104428 | 0.0548776 | 0.033494 | 0.0349349 |
| cg23391288 | 7.48987e-05 | 0.000779174 | 0.000118129 |
| cg23668476 | 0.000463522 | 7.20916e-05 | 2.42747e-05 |
| cg23714751 | 8.75798e-07 | 9.95066e-08 | 2.47243e-07 |
| cg24451839 | 0.124268 | 0.016811 | 0.000477835 |
| cg24475272 | 0.00906028 | 0.0410586 | 0.0138603 |
| cg24533408 | 0.01776 | 0.0156872 | 0.00929792 |
| cg24785495 | 0.00629494 | 0.0519814 | 0.0672238 |
| cg24874433 | 0.0109162 | 0.00353167 | 3.73498e-05 |
| cg24954967 | 0.0100876 | 0.0987285 | 0.0585253 |
| cg25026693 | 0.0144528 | 0.137205 | 0.00816496 |
| cg25300481 | 0.0190966 | 0.00679987 | 0.000711322 |
| cg25551043 | 0.468787 | 0.19635 | 0.119688 |
| cg25699964 | 0.000312609 | 0.0037643 | 0.012723 |
| cg25808826 | 0.0306818 | 0.0108775 | 0.00383221 |
| cg26082814 | 0.0594292 | 0.0315079 | 0.0229628 |
| cg26179530 | 0.0096249 | 0.00831524 | 0.00281571 |
| cg26308668 | 0.000988289 | 0.000196874 | 1.40816e-05 |
| cg26683792 | 0.0047517 | 0.00539723 | 8.97654e-05 |
| cg26685998 | 0.0893609 | 0.00770754 | 0.00734709 |
| cg26760894 | 0.00112045 | 0.015719 | 0.00716435 |
| cg27215601 | 0.0189763 | 0.000600547 | 6.89309e-06 |
| cg27229520 | 0.000325122 | 2.93375e-06 | 8.12215e-08 |
| cg27518631 | 0.012893 | 0.0019512 | 2.6803e-05 |

DNA-methylation levels of the 147 CpG-sites that were not yet differentially methylated in patients upon PICU admission versus healthy children were compared for patients at day 3, day 5 and day 7 in PICU versus healthy children, with use of ANOVA and application of a false discovery rate (FDR) of 0.05. Unadjusted P-values for these comparisons are shown and indicated in yellow when the CpG site was withheld as statistically significantly different between patients and healthy controls after FDR correction at the different time points. All analyses were performed with the Partek Genomics Suite® 7.0 (Partek, St. Louis, MO) software.
