# Supplementary material for: Time course of altered DNA methylation evoked by critical illness and by early administration of parenteral nutrition in the paediatric ICU
Source: Clin Epigenetics. 2020 Oct 20;12:155. doi: 10.1186/s13148-020-00947-w (PMC7576729; doi:10.1186/s13148-020-00947-w)
Supplement: Supplementary file 6 — Additional file 6. Evolution of DNA methylation over time of pattern 1–4 in individual critically ill patients. Detailed time profiles are shown for ten patients of representative CpG-sites classified to pattern 1, pattern 2, pattern 3, and pattern 4. [file 13148_2020_947_MOESM6_ESM.docx]

**Additional file 6. Evolution of DNA-methylation over time of pattern 1-4 in individual critically ill patients.**

Detailed time profiles of representative CpG-sites classified to pattern 1, pattern 2, pattern 3, and pattern 4 are shown for 10 patients. For each CpG-site, we selected 3 patients with samples available on PICU admission, day 3 and last PICU day, 4 patients with samples available on PICU admission, day 3, day 5 and last PICU day, and 3 patients with samples available on PICU admission, day 3, day 5, day 7 and last PICU day. Each coloured line represents one patient. Dots represent the available samples of the patients on the different time points, i.e. if no dot is shown for ∆d5 or ∆d7 this means that the patient had a PICU stay of shorter than 5 or 7 days, respectively. ∆d0: β-value day 0 minus β-value day 0, ∆d3: β-value day 3 minus β-value day 0, ∆d5: β-value day 5 minus β-value day 0, ∆d7: β-value day 7 minus β-value day 0, ∆Ld: β-value last PICU day minus β-value day 0.
