# Supplementary material for: Time course of altered DNA methylation evoked by critical illness and by early administration of parenteral nutrition in the paediatric ICU
Source: Clin Epigenetics. 2020 Oct 20;12:155. doi: 10.1186/s13148-020-00947-w (PMC7576729; doi:10.1186/s13148-020-00947-w)
Supplement: Supplementary file 7 — Additional file 7. Heatmaps of changes in methylation status of all studied CpG-sites of patterns 1–4. Heatmaps summarising the changes in methylation status of the CpG-sites classified to pattern 1, pattern 2, pattern 3 and pattern 4. [file 13148_2020_947_MOESM7_ESM.docx]

**Additional file 7. Heatmaps of all studied CpG sites of pattern 1 to 4.**

Rows indicate the studied CpG-sites. The first column of each heatmap indicates the change in DNA methylation status from PICU admission to day 3 (“∆d3”) and the second column from admission to PICU discharge (“∆Ld”). Shades of red indicate a demethylation from admission to the studied time point and shades of green a hypermethylation, with different shades representing absolute changes in β-values between two time points.
