# Supplementary material for: Time course of altered DNA methylation evoked by critical illness and by early administration of parenteral nutrition in the paediatric ICU
Source: Clin Epigenetics. 2020 Oct 20;12:155. doi: 10.1186/s13148-020-00947-w (PMC7576729; doi:10.1186/s13148-020-00947-w)
Supplement: Supplementary file 8 — Additional file 8. Identification of CpG-sites differentially methylated in patients upon PICU admission as compared with healthy children. DNA methylation levels of patients upon PICU admission were compared with those of healthy children with the use of ANOVA and application of a false discovery rate of 0.05. Unadjusted p values for the 12 CpG-sites already differentially methylated in patients upon PICU admission versus healthy controls are shown. [file 13148_2020_947_MOESM8_ESM.docx]

**Additional file 8. Identification of CpG-sites differentially methylated in patients upon PICU admission as compared with healthy children.**

| **CpG sites** | **Patients upon PICU admission vs healthy children**  **Unadjusted p-values** |
| --- | --- |
| cg01474011 | 3.42523e-16 |
| cg04339882 | 0.00162101 |
| cg04483721 | 3.90251e-06 |
| cg04500577 | 8.76088e-05 |
| cg14772955 | 1.86471e-14 |
| cg15848350 | 1.33717e-09 |
| cg18674312 | 3.21742e-08 |
| cg23313725 | 0.000999756 |
| cg23897894 | 0.000114668 |
| cg25141485 | 3.07102e-05 |
| cg26131710 | 5.65319e-05 |
| cg26434487 | 0.00112682 |

DNA-methylation levels were compared between patients and healthy children with use of ANOVA and applying a false discovery rate (FDR) of 0.05. Unadjusted P-values are shown for the 12 CpG-sites already differentially methylated in patients upon PICU admission versus healthy controls in the current subset. All analyses were performed with the Partek Genomics Suite® 7.0 (Partek, St. Louis, MO) software.
